# ASIA case after injection of liquid silicone^☆^^☆☆^

**DOI:** 10.1016/j.abd.2019.12.006

**Published:** 2020-05-11

**Authors:** Camila Secco Libardi, Lucia Martins Diniz, Carlos Musso, Bruna Anjos Badaró

**Affiliations:** a Sector of Dermatology, Departamento de Clínica Médica, Centro de Ciências da Saúde, Universidade Federal do Espírito Santo, Vitória, ES, Brazil; b Department of Pathology, Centro de Ciências da Saúde, Universidade Federal do Espírito Santo, Vitória, ES, Brazil

Dear Editor,

The appreciation for physical beauty goes back to ancient times. The 20th century brought great changes, with its demanding socioeconomic requirements, influenced by the industrial and petrochemical revolutions, in addition to the ancient human passion: the search for aesthetic perfection.

In 1948, silicone, considered immunologically inert, aroused the interest of the medical community due to the need for biocompatible surgical materials,^1^ gaining importance in cosmiatry. However, from the 1990s, cases of connective tissue disease began to appear in patients with silicone implants, exhibiting fibrous tissue reactions similar to scleroderma. The relationship between silicone implants and appearance of nonspecific symptoms that do not meet diagnostic criteria for connective tissue disease suggests that an undefined syndrome may occur due to silicone exposure.^2^

The analyses of these manifestations led to the definition known as siliconosis, which includes the following: myalgia; abnormal fatigue; cognitive impairment; depression; dry eyes and mouth; skin abnormalities; paresthesias; oedema and sensitivity in axillary glands; fever of undetermined origin; alopecia; headache; morning stiffness.^2^

In recent years, in addition to siliconosis, three other diseases characterized by overactive immune responses have been described, namely: Gulf War syndrome, macrophagic myofasciitis, and post-vaccination phenomena. Since these diseases share a similar set of signs and symptoms, they were condensed by Shoenfeld and Agmon-Levin in 2011 under the term ASIA: autoimmune/inflammatory syndrome induced by adjuvants.^2^ By definition, a case of ASIA characterizes symptoms and systemic signs or autoimmune disease, developed after exposure to external stimuli, with production of antibodies against the adjuvant involved. The diagnostic criteria are listed in table 1.^2^ These criteria have not yet been validated; therefore, there is no consensus in the literature about how many of them should be present for the diagnosis of ASIA. In their cohort, Watada et al. included patients with at least one major, or one major and one minor criteria as cases of the syndrome.^3^

**Table 1:** ASIA diagnostic criteria. Adapted from Shoenfeld & Agmon-Levin, 2011.

ABD suggested criteria for ASIA diagnosis
Major criteria
Exposure to external stimuli (infection, vaccine, silicone, adjuvant) prior to clinical manifestations.
Appearance of “typical” clinical manifestations:
Myalgia, myositis or muscle weakness;
Arthralgia and/or arthritis;
Chronic fatigue, non-restorative sleep or sleep disorders;
Neurological manifestations (especially those related to demyelination);
Cognitive abnormalities, memory loss;
Fever, dry mouth.
Removal of the triggering agent induces clinical improvement.
Typical biopsy of the affected organs.
Minor criteria
Appearance of autoantibodies or antibodies directed against the suspect adjuvant
Other clinical manifestations (e.g., irritable bowel syndrome)
Specific HLA (HLA DRB1, HLA DQB1)
Development of autoimmune disease

In the present case, a 49-year-old woman had received injections of liquid silicone into the buttocks ten years before seeking care. Painful, brownish, hard plaques had appeared on the lateral aspect of the left thigh two years previously (Fig. 1). The lesions evolved into spontaneous outbreaks and remissions, and emerged concurrently with inflammatory arthralgia and asthenia of the hand and wrist. Complementary exams indicated rheumatoid factor 523, ANA 1:80 nuclear fine speckled pattern (remaining rheumatologic panel indicated normal results), corroborating the diagnosis of rheumatoid arthritis. Histopathological analysis of cutaneous lesion indicated chronic inflammatory reaction with xanthomized histiocytes (Fig. 2) and negative acid-fast bacillus (AFB) smears. Treatment with methotrexate was started, controlling the cutaneous lesions and the joint condition.

**Figure 1:**
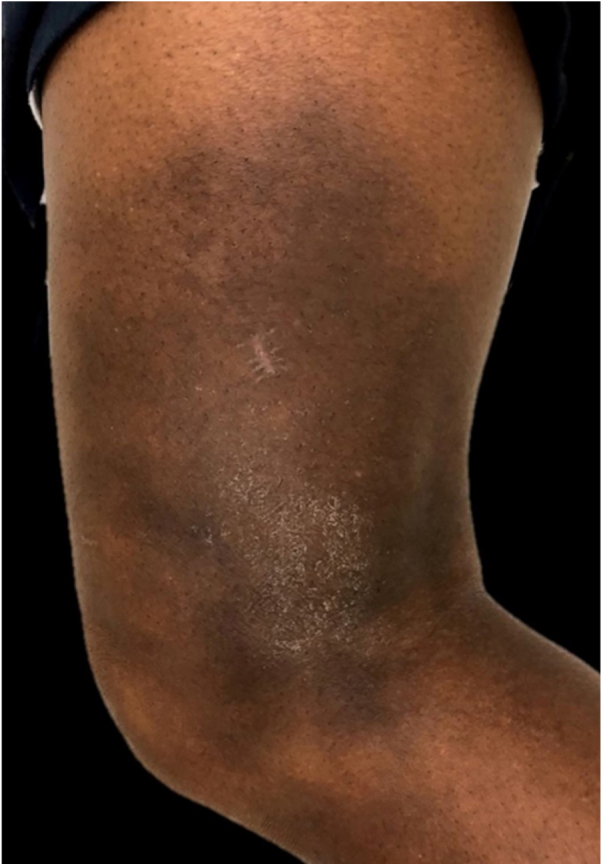
Lateral view of left thigh. Hardened, brownish, scaly plaque.

**Figure 2:**
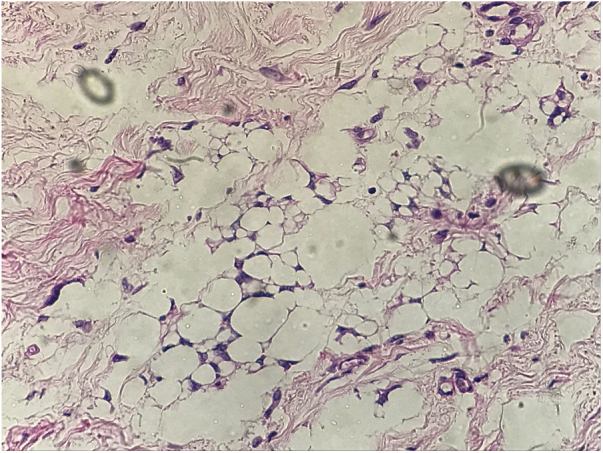
Histopathologic study. Histopathological examination shows xanthomized histiocytes, suggesting reaction to a foreign body.

This report presents three major criteria (silicon exposure; systemic symptoms of asthenia and arthralgias; histopathological exam indicating chronic inflammation), and two minor criteria (onset of rheumatoid arthritis and autoantibodies). Thus, it met the criteria for the diagnosis of the syndrome, which has an uncertain prognosis due to the fact that it is a relatively new concept.

Vera-Lastra et al. analyzed 50 cases of patients submitted to injections of various materials. Inclusion criteria were the following: history of injectable procedures; autoimmune disease/nonspecific manifestations; autoantibodies; histological evidence of chronic inflammation; and absence of infections/neoplasms that explained the picture. The average time between the injections and the onset of the symptoms was around four years (one month to 15 years), with 60% of the patients exhibiting nonspecific autoimmune manifestations, and 8% developing rheumatoid arthritis. According to the histological exam, there were granulomas, chronic inflammation, oil vacuoles, and fibrosis in the injection area,^4^ *i.e.*, findings similar to those of the present case.

Regarding management, Tervaert et al. suggested the following: correction of hypovitaminosis D, if present; reduction of exposure to triggers such as allergic reactions and respiratory tract infections; and smoking cessation. When possible, the involved adjuvant should be surgically removed. In addition, if necessary, medications such as oral or intravenous doxycycline, minocycline, hydroxychloroquine, and corticosteroids should be prescribed.^5^

The present case demonstrates that it is essential to know the patient's symptoms and comorbidities before the injection of adjuvant substances, in order to determine and recognize ASIA, a recently defined syndrome that will have a very persistent future, given the growing demand for injectable procedures. Aware of these data, physicians will be able to avoid the onset of chronic diseases with great impact, which might adversely affect patient's health. Moreover, one more question remains: What other materials, now considered biocompatible (as silicone had once been), could trigger ASIA in the near future? Prospective and retrospective studies could illustrate the ASIA phenomenon within the next decades, providing answers and, possibly, further questions.

## Financial support

None declared.

## Authors’ contributions

Camila Secco Libardi: Conception and planning of the study; drafting and editing of the manuscript; critical review of the literature.

Lucia Martins Diniz: Approval of final version of the manuscript; conception and planning of the study; intellectual participation in the propaedeutic and/or therapeutic conduct of the studied cases; critical review of the manuscript.

Carlos Musso: Approval of final version of the manuscript; collection, analysis, and interpretation of data; critical review of the manuscript.

Bruna Anjos Badaró: Conception and planning of the study; collection, analysis, and interpretation of data; intellectual participation in the propaedeutic and/or therapeutic conduct of the studied cases.

## Conflicts of interest

None declared.
